# The association between polygenic scores for attention‐deficit/hyperactivity disorder and school performance: The role of attention‐deficit/hyperactivity disorder symptoms, polygenic scores for educational attainment, and shared familial factors

**DOI:** 10.1002/jcv2.12030

**Published:** 2021-09-08

**Authors:** Andreas Jangmo, Isabell Brikell, Ralf Kuja‐Halkola, Inna Feldman, Sebastian Lundström, Catarina Almqvist, Cynthia M. Bulik, Henrik Larsson

**Affiliations:** ^1^ Department of Medical Epidemiology and Biostatistics Karolinska Institutet Stockholm Sweden; ^2^ Department of Economics and Business Economics National Centre for Register‐Based Research Aarhus University Aarhus Denmark; ^3^ Department of Public Health and Caring Sciences Uppsala University Uppsala Sweden; ^4^ Gillberg Neuropsychiatry Centre Institute of Neuroscience and Physiology Sahlgrenska Academy University of Gothenburg Sweden; ^5^ Centre for Ethics Law and Mental Health (CELAM) Institute of Neuroscience and Physiology Sahlgrenska Academy University of Gothenburg Sweden; ^6^ Astrid Lindgren Children's Hospital Karolinska University Hospital Stockholm Sweden; ^7^ Department of Psychiatry University of North Carolina at Chapel Hill Chapel Hill North Carolina USA; ^8^ Department of Nutrition University of North Carolina at Chapel Hill Chapel Hill North Carolina USA; ^9^ School of Medical Sciences Örebro University Örebro Sweden

**Keywords:** ADHD, polygenic scores, school performance

## Abstract

**Background:**

Polygenic scores (PGS) for attention‐deficit/hyperactivity disorder (ADHD) negatively predicts educational attainment (EA), but it remains unclear how ADHD symptoms, PGS for EA, and shared familiar factors influence the associations between PGS for ADHD and school performance.

**Method:**

We combined survey data on ADHD symptoms, PGS, and register‐based, objective measures of compulsory school performance at age 16 for 6049 twins in the Child and Adolescent Twin Study in Sweden. Linear and instrumental variable (IV) regression models were used to estimate the association between PGS for ADHD and grade point average (GPA), overall and by natural science, humanities, and practically oriented (e.g., sports, arts, music) subject categories. The models were adjusted for parent‐rated ADHD symptoms, PGS for EA, and shared familial factors (dizygotic twin comparisons) to examine how these factors influenced the associations between PGS for ADHD and school performance.

**Results:**

PGS for ADHD were negatively associated with school performance; *β* = −0.12, 95% confidence interval = (−0.15, −0.09) for overall GPA with minor differences by subject category. Adjustment for ADHD symptoms attenuated these associations to a small degree compared to PGS for EA, and shared familial factors respectively. Stronger associations were observed using IV regressions compared to linear regression. However, in the IV regression analyses, most associations between PGS for ADHD and GPA in the practically oriented subject category were not significant.

**Conclusion:**

Associations between PGS for ADHD and school performance are to a small degree influenced by ADHD symptoms, compared to PGS for EA and shared familial factors. These results highlight important considerations for research using PGS for ADHD to control for genetic factors, and for future clinical applications aiming to determine genetic liability towards ADHD.

**Key points**

- Polygenic scores for ADHD are negatively associated with a broad range of educational outcomes.
- The association between polygenic scores for ADHD and school performance is to a small degree explained by phenotypic ADHD symptoms.
- Polygenic scores for educational attainment and, in particular, shared familial factors are of high importance in the association between polygenic scores for ADHD and school performance.
- The weak influence of phenotypic ADHD symptoms on these associations highlight important considerations in research and clinical applications regarding the specificity of polygenic scores for ADHD.

## INTRODUCTION

Attention‐deficit/hyperactivity disorder (ADHD) is characterized by impairing and developmentally age‐inappropriate inattentive and hyperactive behaviors (American Psychiatric Association, 2013). Individuals with ADHD often experience academic difficulties (e.g., failing grades; Jangmo et al., 2019), and poor occupational outcomes in adulthood (Gordon & Fabiano, 2019). This highlights the need to understand the etiology of educational outcomes in ADHD.

Genetic factors are important in ADHD, with heritability estimates around 70%–80% (Faraone & Larsson, 2018), and 25% in Genome‐Wide Association studies (GWAS; Demontis et al., 2019). Educational outcomes (e.g., years of education, school grades) also show substantial genetic influence (Lee et al., 2018; Shakeshaft et al., 2013), and twin and GWAS based genetic correlations between ADHD and educational outcomes to around −0.3 to −0.5 (Daucourt et al., 2020; Demontis et al., 2019; Greven et al., 2014). One study found that 30 out of 58 GWAS loci associated with ADHD were shared with loci for educational attainment (EA) or intelligence (O'Connell et al., 2019). Research using polygenic scores (PGS), constructed by summing an individual's effect alleles multiplied by effect sizes estimated in GWAS (Martin et al., 2019), have found that PGS for EA in adults predicts ADHD and test scores in children (de Zeeuw et al., 2014). Likewise, PGS for ADHD are negatively associated with educational and cognitive outcomes, and school test scores (Selzam et al., 2019; Stergiakouli et al., 2016). However, one study found that ADHD symptoms only mediates 16% of the association between PGS for ADHD and school performance (Stergiakouli et al., 2016). The availability of PGS from larger ADHD GWAS (Demontis et al., 2019), makes it relevant to re‐examine how phenotypic ADHD symptoms influence the associations between PGS for ADHD and educational outcomes.

Interpreting observed associations between PGS for ADHD and educational outcomes is challenging. Findings may reflect pleiotropy (i.e., that the same genetic variants affects several traits), linkage disequillibrium (i.e., genetic variants inherited together), and environmental or diagnostic mechanisms (e.g., diagnostic misclassification) (Morris et al., 2020; van Rheenen et al., 2019). For example, are associations between PGS for ADHD and cognitive tasks (e.g., verbal reasoning), other psychiatric and somatic disorders (Du Rietz et al., 2018; Jansen et al., 2019; Li, 2019) unique to ADHD or due to the genetic overlap between ADHD and EA? PGS for EA correlate with factors like smoking during pregnancy and parental education (Krapohl et al., 2017), treatment‐seeking behaviors are genetically correlated with EA, ADHD, and other psychiatric disorders (Rayner et al., 2019). Furthermore, parental non‐transmitted alleles (i.e., genetic variants present in parents but not in offspring) associates with offspring educational outcomes (genetic nurturing) (Kong et al., 2018; de Zeeuw et al., 2020). Hypothetically, genetic variants associated with clinically diagnosed ADHD may correlate with other genetically influenced traits that are more prevalent in clinical populations (e.g., through functional impairments), compared to the population below the clinical diagnostic threshold (Morris et al., 2020). Studies accounting for factors shared by family members have found that associations attenuated by about 40% when comparing siblings in the largest GWAS of EA (Lee et al., 2018), and attenuated associations between PGS for ADHD and standardized tests (*N* = 2366 twin pairs) (Selzam et al., 2019). Shared familial factors beyond individual genetics, may thus explain a significant portion of the associations between PGS for ADHD and educational outcomes.

Other limitations remain unaddressed regarding the polygenic influence of ADHD on educational outcomes. School performance has mainly been evaluated by mathematics and language tests. Grades may capture school performance more broadly, as teachers evaluate student performance across different dimensions (Brookhart et al., 2016). Moreover, genetic factors affect both subject choice, performance (Rimfeld et al., 2016), and ADHD symptoms have been positively associated with choosing sport educational university programs (Gökçen et al., 2013). Thus, variability in associations between PGS for ADHD and subject type is plausible but untested. One twin study found similar genetic correlations between achievements across different subjects (e.g., arts, science, humanities) (Rimfeld et al., 2015), but whether such patterns extend to PGS for ADHD is unknown. Lastly, associations between PGS for ADHD and school performance are likely attenuated by measurement error in the PGS. Instrumental variable (IV) methods (e.g., Mendelian randomization; DiPrete et al., 2018) may reduce such problems, but has to our knowledge not been applied to the association between school performance and PGS for ADHD.

Our aim was to examine the influence of ADHD symptoms, PGS for EA, and shared familial factors on the association between PGS for ADHD and school performance, and evaluate whether these associations differed between school subjects. We utilized register‐based school performance measurements among 6049 Swedish twins around age 16, to estimate associations between school performance and PGS for ADHD, and adjusted these associations for ADHD symptoms, PGS for EA, and shared familial factors respectively, both using linear regression (LR) and IV analyses with two different PGS for ADHD (i.e., PGS based on GWAS of clinical ADHD and ADHD symptoms).

## METHODS

### Data sources

We extracted data on 6049 twins from the Child and Adolescent Twin Study in Sweden (CATSS) that have been genotyped. Parents of participants in CATSS were interviewed via telephone regarding behavior, development, and other characteristics at ages 9 or 12 years (Anckarsäter et al., 2011). The twins were individually linked to multiple Swedish national registers using their personal identification number. The National School Register (NSR) contains complete information on leaving certificate grades for all individuals that finish compulsory school in Sweden. Additional information on data sources used for supplementary analyses are presented in the supplementary material. Informed consent for participation in CATSS was obtained from parents, and ethical approval was obtained from the regional ethics board in Stockholm.

#### Genotype data

All dizygotic twin pairs and one twin among monozygotic pairs in the current sample were genotyped using the Illumina Infinium PsychArray‐24 BeadChip as part of a larger sample of twins (*N* = 18,560). Of these, 248 samples identified as non‐European were excluded as the discovery GWAS were based on European samples. Non‐genotyped twins in monozygotic pairs had their genotype inferred from their genotyped co‐twin. Details of genotyping procedure, quality control, imputation, and principal component analysis (PCA) have been published elsewhere (Brikell et al., 2018).

### Definition of ADHD symptoms

Child ADHD symptoms were rated using the A‐TAC questionnaire. A‐TAC contains 10 questions about impulsive behavior and activity level, and nine questions regarding concentration and attention, scored using a three‐level scale: “No” = 0, “Yes, sometimes” = 0.5, and “Yes” = 1. This scale correlates strongly with a diagnosis ofADHD, as indicated by an area under the curve statistic of 0.93 (Mårland et al., 2017). A mean score for each twin was created on A‐TAC's concentration‐attention and impulsiveness‐activity scales separately, and combined.

### Derivation of PGS for ADHD and EA

PGS were calculated based on the most recent GWAS of EA (years of education; *N* = 1.2 million) (Lee et al., 2018), ADHD diagnosis (19,099 cases, 34,194 controls) (Demontis et al., 2019), and population‐based ADHD symptoms (*N* = 17,666) (Middeldorp et al., 2016). Overlapping single nucleotide polymorphisms (SNPs) across discovery samples and the CATSS sample were retained and variants were filtered on minor allele frequency (<0.05 or >0.95), and imputation quality (INFO<0.8). To select a relatively independent set of SNPs for PGS calculation, we ran LD‐clumping (*r* ^2^ < 0.1 in 1 Mb window) on the overlapping SNPs using 1000 Genomes Project European samples as LD‐reference. PGS were calculated for each trait, including SNPs based on seven *p*‐value thresholds (*p* < 0.001, *p* < 0.01, *p* < 0.1, *p* < 0.2, *p* < 0.3, *p* < 0.5, *p* ≤ 1) using PLINK. Finally, we ran a PCA on these PGS within each trait to derive a PGS based on the first principal component. A recent paper has shown that this approach can incorporate the majority of polygenic variability in the PGS (Coombes et al., 2020). Hereafter, we abbreviate our PGS as EA‐PGS, ADHD‐PGS (ADHD diagnosis), and ADHS‐PGS (ADHD symptoms), while “PGS for ADHD” is used when referring to both PGS or such PGS in general.

### Definition of school performance

School performance was indexed by the grade point average (GPA, range 0–20 points), overall, and in three different subject categories defined based on their content: natural science (biology, chemistry, mathematics, physics), humanities/language (civics, English, geography, history, religion, Swedish), and practically oriented (arts, crafts, home and consumer studies, music, sports, technology) subjects. See Appendix S1 for details on grading in Swedish compulsory school, and the calculation of the GPA.

### Covariates

Sex at birth was determined from the Total Population Register (1 when female, 0 when male). Compulsory school graduation year (2008–2013) was determined from the NSR and coded as a continuous variable (0−5).

## ANALYSES

We addressed our aims using both ordinary and IV analyses, and applied these to all school performance measures.

### LR analyses

We estimated a crude association between ADHD‐PGS and school performance using LR. The associations were adjusted for sex, the linear effect of compulsory school graduation year, and five PC's were included to control for potential population stratification (i.e., ancestral genetic differences).

Next, we adjusted these associations for ADHD symptoms, EA‐PGS, ADHD symptoms and EA‐PGS jointly, and shared familial factors in separate models. A family fixed effects approach (i.e., comparing dizygotic twins from the same family) was used to adjust associations for shared familial factors where each twin pair is treated as a separate stratum, thereby removing influences from factors shared between twins from the same family (Gunasekara et al., 2014). Such factors include correlations between the PGS and shared environmental factors, population structure, and others (Selzam et al., 2019). Sex was the only covariate in this analysis.

### IV analyses

**FIGURE 1:**
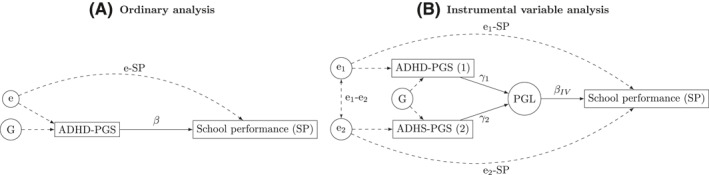
Linear and instrumental variable regression analyses of the association between polygenic scores and school performance. Rectangles represent measured variables, and circles unobserved. Filled lines represent associations possible to estimate using the data available in the current study. Dashed lines represent unobserved associations. (A) Ordinary analysis using either polygenic score (1 or 2). (B) Instrumental variable analysis using both polygenic scores. e, unmeasured influences on PGS and school performance; G, genetic factors; PGL, polygenic load; PGS, polygenic score

We used an IV approach to reduce the influence of measurement error (Figure 1A, path e_1_‐SP) on the association between ADHD‐PGS and school performance (see Appendix S2 for a formal treatment). Multiple measurements of a variable can act as instruments for each other (Wooldridge, 2010), and the ADHD‐PGS and ADHS‐PGS can be considered as imperfect measures of an unobserved polygenic load (Figure 1B, PGL). Given that these PGS weigh effect alleles differently, the IV approach can reduce the impact of measurement error (Figure 1B, paths e_1_‐SP and e_2_‐SP) on the association with school performance as the IV estimate only relies on the PGS mutual dependence on the polygenic load. Key IV assumptions are first that the different PGS for ADHD correlate significantly with each other (Figure 1B); second, that this correlation arises exclusively due to the PGSs mutual dependence on the unobserved polygenic load; and third, that the measurement error in the PGSs are independent of each other (Figure 1B, path e_1_‐e_2_ = 0). We used two‐stage least squares regression was used for estimation; first regressing the ADHD‐PGS on the ADHS‐PGS, then regressing the outcome on the predictions of ADHD‐PGS, including any covariates at both stages. The necessary assumptions were evaluated by the first stage results, rejection of null hypothesis Cov(ADHD‐PGS, ADHS‐PGS) = 0, and while the second assumption is untestable, we replicated previous associations between ADHD‐PGS and phenotypic ADHD symptoms and a clinical diagnosis of ADHD (Aguilar‐Lacasaña et al., 2020; Selzam et al., 2019) in our sample.

### Supplementary analyses

For transparent reporting, consistent with open science principles, and to support future meta‐analytical work, we estimated associations between the ADHD‐PGS and individual subjects. We calculated phenotypic correlations between ADHD and school performance to examine differences by subject category and stratified the ordinary analyses on sex to examine any sex‐dependent associations. To evaluate whether associations differed by ADHD subtypes, analyses were performed separately for concentration‐attention and impulsiveness‐activity symptom scales. We also performed simulations to evaluate how well the LR, and IV analyses performed when the relations between the underlying variables were known.

The R‐package lfe (Gaure, 2013) was used for LR and IV analyses. Outcomes and exposures were standardized to mean zero and standard deviation (SD) one, thus the estimated coefficients reflect associations in terms of SDs. Standard errors in all analyses were clustered on families to account for dependency between observations from the same twin pair.

## RESULTS

Table 1 presents descriptive statistics; 59.8% (*N* = 3620) of twins were dizygotic, with a slightly higher proportion of females among monozygotic twins, 53% versus 47%. Rates of missingness were low (<1%) in GPA and individual subjects (Appendix S1).

**TABLE 1:** Descriptive statistics

	All	Monozygotic sample	Dizygotic sample
*N*	6049	2416 (40.2)	3620 (59.8)
Female	2099 (49.7)	1280 (53.0)	1719 (47.5)
A‐TAC ADHD symptoms^a^	0 (1)	−0.07 (0.91)	0.05 (1.06)
Missing >0 grade in GPA	64 (1.1)	19 (0.8)	43 (1.2)
Polygenic scores			
ADHD symptoms^a^	0.00 (1.00)	−0.01 (0.99)	0.01 (1.00)
ADHD diagnosis^a^	0.00 (1.00)	0.02 (1.00)	−0.02 (1.00)
Educational attainment^a^	0.00 (1.00)	0.00 (1.01)	0.00 (0.99)

*Note*: N (%) unless stated.

Abbreviations: ADHD, attention‐deficit/hyperactivity disorder; GPA, grade point average.

^a^ Mean (standard deviation).

### LR analyses

**FIGURE 2:**
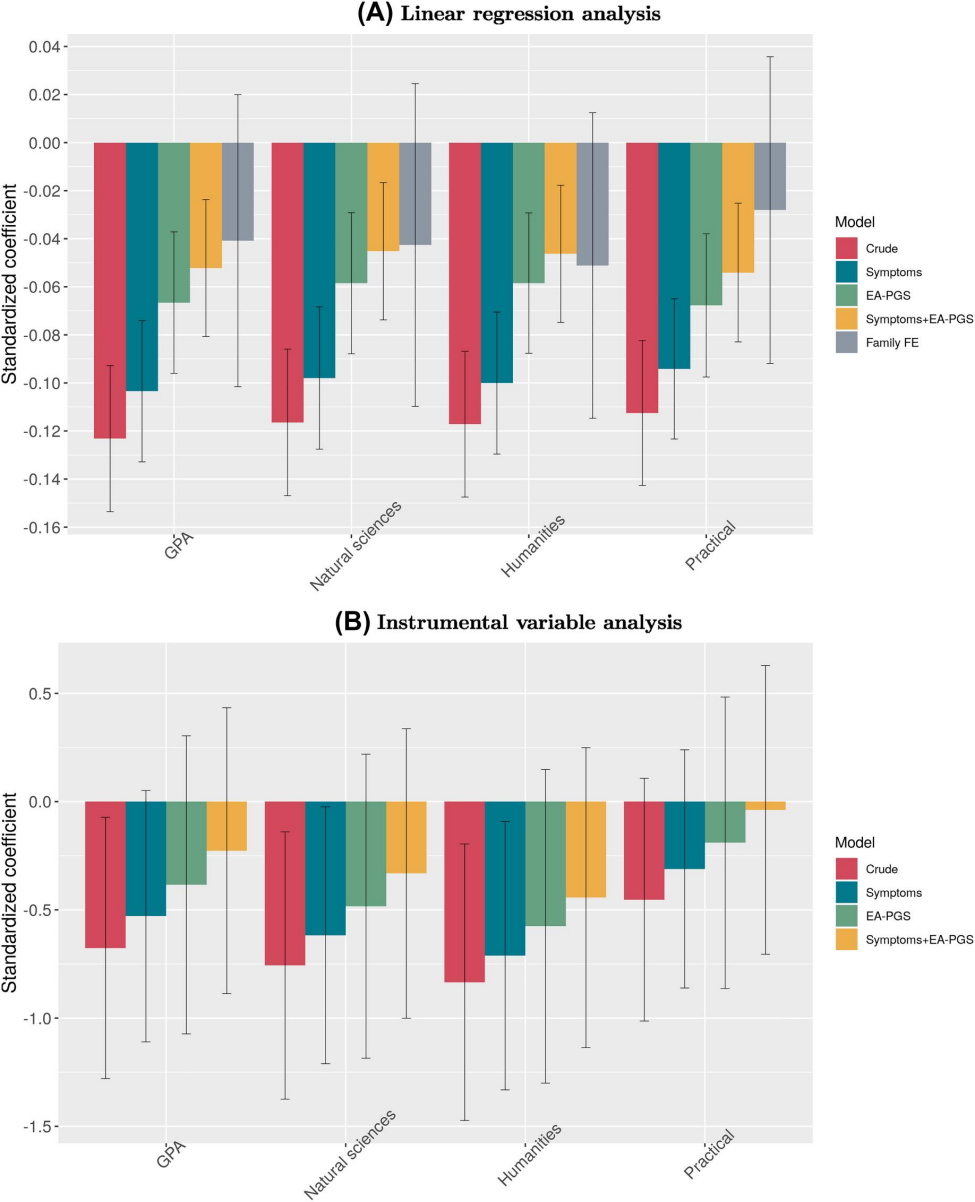
Association between polygenic scores for ADHD and school performance. (A) Linear regression analysis. (B) Instrumental variable analysis. See Table 2 legend and method section for details on the statistical model. Natural sciences: GPA in biology, chemistry, mathematics, and physics. Humanities: GPA in civics, English, geography, history, religion, and Swedish. Practical: GPA in arts, crafts, home and consumer studies, music, sports, and technology. Crude: Model adjusted for sex and the first five principal components. Symptoms: Adjustment for phenotypic ADHD symptoms. EA‐PGS: Model adjusted for PGS for EA. Symptoms + EA‐PGS: Adjustment for phenotypic ADHD symptoms and PGS for EA. Family FE: Adjustement for family fixed‐effects, that is, a dizygotic twin comparison. ADHD, attention‐deficit/hyperactivity disorder; EA‐PGS, polygenic score for educational attainment; GPA, grade point average; PGS, polygenic score

Rows labeled “LR” in Table 2 and Figure 2A presents results from the LR analyses of the ADHD‐PGS and school performance. A one SD increase in the ADHD‐PGS was associated with a *β* = −0.12 (95% confidence interval −0.15, −0.09) SD lower GPA. The association between the ADHD‐PGS and overall GPA decreased to *β* = −0.10 (−0.13, −0.07) when adjusting for ADHD symptoms, and to *β* = −0.07 (−0.10, −0.04) when adjusting for EA‐PGS. Joint adjustment for ADHD symptoms and the EA‐PGS in the same model had no marked influence on the associations. When comparing twins from the same family, the associations dropped to *β* = −0.04 (−0.10, 0.02) (Table 2). The associations between overall GPA and the ADHD‐PGS were generally similar for the natural science, humanities and practical subject categories (Figure 2A).

**TABLE 2:** Associations between polygenic scores for ADHD diagnosis and school performance

			Model adjustments			
		Crude	ADHD symptoms	EA‐PGS	EA‐PGS + ADHD symptoms	Within family
		Standardized coefficient (95% CI); % attenuation				
GPA	LR	−0.12; REF	−0.10; −17%	−0.07; −42%	−0.05; −58%	−0.04; −67%
		(−0.15, −0.09)	(−0.13, −0.07)	(−0.10, −0.04)	(−0.08, −0.02)	(−0.10, 0.02)
	IV	−0.68; REF	−0.53; −22%	−0.38; −59%	−0.23; −66%	NA
		(−1.28, −0.07)	(−1.11, 0.05)	(−1.07, 0.30)	(−0.89, 0.43)

*Note*: The ADHS‐PGS is used as an instrument for the ADHD‐PGS (the exposure). Results from within family IV analyses were omitted due to that the instrument and exposure were not significantly associated (*p* < 0.05) in the first stage IV regressions. % attenuation: The percentage difference between the crude and adjusted point estimates, 100 · (1−*β*_adj_.*/β*_crude_). GPA, ADHD symptoms, and PGS have been standardized to zero mean and unit variance. Between family models are adjusted for female sex, the first five principal components, and the linear effect of graduation year. Within family models are adjusted for sex.

Abbreviations: ADHD, attention‐deficit/hyperactivity disorder; CI, confidence interval; EA‐PGS, polygenic score for educational attainment; GPA, grade point average; LR, linear regression; PGS, polygenic score; IV: Instrumental variable analysis using two‐stage least squares regression.

### IV analyses

#### Evaluation of IV assumptions

The ADHD‐PGS and the ADHS‐PGS correlated significantly (*β* = 0.06; *p* < 0.001; F‐statistic = 15.6), and after adjusting for phenotypic ADHD symptoms, and EA‐PGS, but not when adjusting for shared familial factors (Table S1). A one SD increase in the ADHD‐PGS associated with an *β* = 0.08 SD higher ADHD symptoms using the ordinary analysis, and *β* = 0.63 SDs in the IV analysis. The associations with a clinical diagnosis of ADHD were comparable in the LR and IV analyses (Table S2).

Rows labeled “IV” in Table 2 and Figure 2B presents results from the IV analyses using the ADHS‐PGS as an instrument for the ADHD‐PGS (Figure S1 for results when these roles are reversed). The crude association between the ADHD‐PGS and GPA was estimated to *β* = −0.68 (−1.28, −0.07). Compared to the LR analyses, adjustment for ADHD symptoms, EA‐PGS, and shared familial factors influenced the associations similarly in relative terms (Table 2). But the association between ADHD‐PGS and practical subject GPA was weaker relative to associations with natural science and humanities (Figure 2).

### Supplementary analyses

The simulated IV analyses showed that the mean estimates were closer to the target association than LR, but the IV estimates were much more variable which highlights uncertainties regarding the strength of the polygenic influence (Appendix S2). The association between ADHD‐PGS and GPA was slightly weaker among females than males (Figure S2), and no marked differences in terms of association between the ADHD‐PGS and GPA were found when attention‐concentration and impulsiveness‐activity symptom scales were analyzed separately compared to total ADHD symptoms (Figure S3).

## DISCUSSION

We identified important associations between PGS for ADHD and register‐based measures of school performance among 6049 Swedish twins. The ADHD‐PGS associated negatively with GPA, overall, and in different subject categories. ADHD symptoms influenced these associations minimally compared to the more pronounced influence from EA‐PGS, and the substantial influence from shared familial factors when comparing dizygotic twins. Using multiple PGS for ADHD in an IV analysis, we found a potentially weaker polygenic overlap between ADHD and more practically oriented subjects than others. To our knowledge, this is a novel IV analysis of PGS for ADHD. But given the methodological assumptions, and uncertain estimates (wide confidence intervals), these findings should be verified by other studies.

We found that adjustment for shared familial factors attenuated the associations between ADHD‐PGS and school performance by about 67%, suggesting a stronger influence compared to previous research on 2366 twin pairs (Selzam et al., 2019). This may be explained by that we modeled shared familial factors as a fixed‐effect, while the former study used random‐effects. Random‐effects models may be biased if the random effect (the shared familial factors) is correlated with the exposure (the child PGS) (Gunasekara et al., 2014). Our results may thus implicate a stronger role for genetic and environmentally shared familial factors in the relation between PGS for ADHD and school performance. Environmentally shared familial factors may include the positive association between ADHD and lower parental socioeconomic status (Russell et al., 2016), which may be elevated in the presence of a parental history of ADHD (Rowland et al., 2018). Given the positive relation between higher parental socioeconomic status and school performance in offspring (von Stumm et al., 2020), such associations may represent a combination between genetically and environmentally conferred risk for both ADHD and school performance. Targeted interventions to manage such factors may thus reduce the negative influence from the polygenic load of ADHD on school performance. Our results also have implications for research either using PGS for ADHD to control for genetic factors, or to understand the relationship between genetic factors for ADHD and other outcomes. Given the widespread pleiotropy of PGS for EA, our finding that PGS for ADHD appear to capture more components of PGS for EA than ADHD symptoms, may mean that PGS for ADHD represent polygenic influence from both components and consequences of ADHD, along with comorbid, and environmental factors that mediate polygenic influences of ADHD on school performance. Research utilizing PGS for ADHD to control for polygenic influences from ADHD should be cautious regarding the specificity of these PGS to ADHD. From a clinical perspective, these results further emphasize limitations in the ability of PGS for ADHD to discriminate between cases and non‐cases.

The crude analyses of school performance revealed that the ADHD‐PGS associated negatively with GPA, explaining about 1.5% of GPA variation, and was similar for different subjects (Figure 2A and Figure S4). Previous research has found that PGS for ADHD explain about 0.6% (*R* ^2^ = 0.006) of variation in test results (age 16) (Stergiakouli et al., 2016), and 1% in literacy/language related abilities (age 7–13) (Verhoef et al., 2019). Adjusting for phenotypic ADHD symptoms attenuated all associations between the ADHD‐PGS and school performance, but confidence intervals overlapped with crude associations. We confirm prior findings that ADHD symptoms only mediates 16% of the association between PGS for ADHD and school performance (Stergiakouli et al., 2016). That adjustment for EA‐PGS attenuated the associations more (42%) may be explained by the genetic overlap between ADHD and EA (O'Connell et al., 2019).

When we utilized PGS based on ADHD symptoms and clinical diagnosis in an IV analysis, we found a stronger polygenic influence of ADHD on school performance compared to LR. A Mendelian randomization study found similar associations between ADHD and language and literacy related abilities, but concluded that IV assumptions were violated (Verhoef et al., 2019), and similar limitations likely apply to our results. Still, compared to the LR analyses, the crude IV association with the practical subject category was almost half of that in other categories (Figure 2B, Figure S4 for individual subjects). One explanation may be pleiotropic influences from genetic factors on different school subjects (Rimfeld et al., 2015) and the positive correlation between PGS for EA and a latent factor of school performance (Rajagopal et al., 2020). Hypothetically, the IV analysis decreases the sensitivity of the PGS towards this latent factor, and increases specificity towards polygenic influences from ADHD to school performance. Notably, differences between subject category GPA and overall GPA (e.g., subtracting overall GPA from natural science GPA) is apparent in both LR and IV analyses, but the relative influence from ADHD symptoms and EA‐PGS on these associations appears lower in the IV analysis (Figure S5).

Lastly, it should be noted that ADHD symptoms and severity can vary over time, and that functioning in ADHD is impacted by psychiatric comorbidities that also associate negatively with school performance (e.g., anxiety and depression) (Dalsgaard et al., 2020). Future research should therefore consider variation in ADHD symptoms as well as correlated psychopathology at different timepoints across development.

### Strengths and limitations

Strengths include the use of register‐based school performance measurements in a large twin sample. With regards to external validity, the response rate in CATSS is high (75%) with small differences between responders and non‐responders in terms of variables such as socioeconomic status (Anckarsäter et al., 2011). We used a validated measure of ADHD symptoms (A‐TAC) (Mårland et al., 2017), which correlated similarly with PGS for ADHD as a recent study using the SWAN scale (Burton et al., 2019).

Limitations include the low predictive ability of the ADHD‐PGS, and the uncertain estimates from the IV analyses (wide confidence intervals). However, we have emphasized general tendencies, rather than outcomes from significance testing. Future studies using larger samples are needed to confirm our findings. While register‐based measurements of school performance has advantages compared to survey based measures (e.g., no recall bias, low attrition), we acknowledged that these benefits do not circumvent biases in how teachers grade students (e.g., classroom behavior) that may affect our estimates, in particular when adjusting for phenotypic ADHD symptoms. We also acknowledge that more recent methods to calculate PGS (e.g., LDpred) (Privé et al., 2021) are available than the thresholding and clumping approach we used. Thus, we were limited to the older method because at the time the PGS were calculated and linked to the register‐based measures, the newer approach was not available.

## CONCLUSION

Currently available PGS for ADHD associate negatively with school performance at age 16, and these associations are similar across different school subjects. IV analyses suggest weaker associations between the ADHD polygenic load and more practical subjects (e.g., sport, arts) than others. Overall, these associations are influenced to a small extent by phenotypic ADHD symptoms, to a greater degree by PGS for EA, while shared familial factors show the strongest influence.

## CONFLICTS OF INTEREST

Andreas Jangmo has served as a speaker for Takeda. Cynthia M. Bulik reports: Shire/Takeda (grant recipient, Scientific Advisory Board member) and is on the Editorial Advisory Board for JCPP *Advances*; Pearson (author, royalty recipient); Henrik Larsson has served as a speaker for Evolan Pharma and Takeda/Shire, and has received research grants from Takeda/Shire, all outside the submitted work. He is Editor‐in‐Chief for JCPP *Advances*. [Corrections made on 22 June 2022, after first online publication: This Conflicts of Interest statement has been updated in this version.]

## ETHICAL APPROVAL

Informed consent for participation in CATSS was obtained from parents, and ethical approval was obtained from the Regional Ethics Board in Stockholm.

## AUTHOR CONTRIBUTIONS

Andreas Jangmo: Manuscript, original draft and review/editing, formal analysis, methodology, visualization. Isabell Brikell: Manuscript, original draft and review/editing. Ralf Kuja‐Halkola: Manuscript, original draft and review/editing. Inna Feldman: Manuscript, original draft and review/editing. Sebastian Lundström: Manuscript, original draft and review/editing. Catarina Almqvist: Manuscript, original draft and review/editing. Cynthia M. Bulik: Manuscript, original draft and review/editing. Henrik Larsson: Manuscript, original draft and review/editing, conceptualization, funding acquisition, supervision.

## Supporting information

Supporting Information 1

## Data Availability

Due to legal restrictions in the Swedish Secrecy Act, we are unable to share even de‐identified data. The data used in this study was obtained from the national Swedish registers and made available after ethical approval. The registers used for this study include: National Patient Register, Multi‐Generation Register, Prescribed Drug Register, and the Swedish Twin Registers. Researchers may apply for access these data sources through the Swedish Ethical Review Authority (etikprovningsmyndigheten.se; registrator@etikprovning.se) and from the primary data owners: Statistics Sweden (scb@scb.se), the National Board of Health and Welfare (socialstyrelsen@socialstyrelsen.se), and the Swedish Twin Register (str‐research@meb.ki.se), in accordance with Swedish law.
