# Supplementary material for: The association between polygenic scores for attention‐deficit/hyperactivity disorder and school performance: The role of attention‐deficit/hyperactivity disorder symptoms, polygenic scores for educational attainment, and shared familial factors
Source: JCPP Adv. 2021 Sep 8;1(3):e12030. doi: 10.1002/jcv2.12030 (PMC10242908; doi:10.1002/jcv2.12030)
Supplement: Supplementary file 1 — Supporting Information 1 [file JCV2-1-e12030-s001.docx]

**The association between polygenic scores for ADHD and school performance: The role of ADHD symptoms, polygenic scores for educational attainment, and shared familial factors: Supplementary material**

Andreas Jangmo, MSc^1^; Isabell Brikell, PhD^1^; Ralf Kuja-Halkola, PhD^1^; Inna Feldman, PhD^2^; Sebastian Lundström, PhD^3,4^; Catarina Almqvist, MD, PhD^1,5^; Cynthia M. Bulik, PhD^1,6,7^; Henrik Larsson, PhD^1,8^ .

1 Department of Medical Epidemiology and Biostatistics, Karolinska Institutet, Stockholm, Sweden.

2 Department of Public Health and Caring Sciences, Uppsala University, Uppsala, Sweden.

3 Gillberg Neuropsychiatry Centre, Institute of Neuroscience and Physiology, Sahlgrenska Academy, University

of Gothenburg, Sweden.

4 Centre for Ethics Law and Mental Health (CELAM), Institute of Neuroscience and Physiology, Sahlgrenska

Academy, University of Gothenburg, Sweden.

5 Astrid Lindgren Children’s Hospital, Karolinska University Hospital, Stockholm, Sweden.

6 Department of Psychiatry, University of North Carolina at Chapel Hill, Chapel Hill, North Carolina, USA.

7 Department of Nutrition, University of North Carolina at Chapel Hill, Chapel Hill, North Carolina, USA.

8 School of Medical Sciences, Örebro University, Örebro, Sweden.

**Appendix S1: Description of the Swedish school system and subjects included in the grade point average**

Swedish compulsory school is 9 years in length and leaving certificate grades are awarded at the end of the 9th year. These determine student’s options when applying for further studies in upper secondary school. When the teacher in each subject awards the grade, the students overall performance should be evaluated against the nationally set goals in each subject. For Swedish, English, and mathematics (the core subjects which students are required to pass) the teacher is also aided by national standardized tests that take place in the final semester (1).

Students were graded on a four point alphabetical scale between 1998 and 2012, and a 6 point scale from 2013. We converted the alphabetical grades according to the official conversion provided by the Swedish National Agency for Education.^[[1]](#footnote-1)^

Approximately 25 percent of the students in the current study received an average grade in at least one out of two groups of subjects. These groups consisted of natural science oriented subjects (biology, chemistry, and physics), and humanities oriented subjects (geography, civics, history, and religion). To simplify the analysis and maximize the sample size, we averaged the individual grades in these subject groups for the students that did not receive these grouped grades. Missing subjects grades were omitted in the calculation of the GPA. Rates of missingness for the outcomes were as follows

| Outcome | Percent missing |
| --- | --- |
| GPA | 0.5 |
| *Natural sciences* | |
| Mathematics | 0.6 |
| Bio + Che + Phy | 0.7 |
| *Humanities* | |
| English | 0.7 |
| Swedish | 0.6 |
| Civ + Geo + His + Rel | 0.6 |
| *Practical* | |
| Arts | 0.7 |
| Crafts | 0.8 |
| Home/Consumer studies | 0.7 |
| Music | 0.7 |
| Sports | 0.7 |
| Technology | 0.7 |
| Civ+Geo+His+Rel: Civics, geography, history, and religion.  Bio+Che+Phy: Biology, chemistry, and physics. | |

**Appendix S2: Instrumental variable analysis using multiple polygenic scores for ADHD**

**The model**

The outline here builds upon models as described in (2). See (3) for an application to genome-wide association studies.

Denote a polygenic score (PGS) for ADHD with A G and the unobserved polygenic load with A. The PGS for ADHD correlates with this load with an error:

$A_{G}=bA+e$(1)

The target association to estimate is β in:

$y=\alpha+\beta A+\varepsilon$(2)

But since we only have access to an imperfect measure of A, we rearrange this equation to $A=\left( A_{G}-e \right)/b$. Regressing A_G_ on an outcome y will lead to an attenuation in the coefficient:

$y=\alpha+\gamma\left( A_{G}-e \right)+\varepsilon$(3)

where γ= β/b. Note that if b = 1, then A is perfectly measured by A_G_, and thus e = 0.

Since the measurement error e is unobserved, the estimated association between the polygenic load for ADHD and y will be attenuated due to the correlation between γ and the measurement error (-γe). However, if there is another measure of A, utilizing both of them can compensate for the attenuation. Define two PGS, A_G,1_ and A_G,2_ that both measure A with an error:

$A_{G,1}=b_{1A}+e_{1}$(4)

$A_{G,2}=b_{2A}+e_{2}$(5)

Rewrite (4) in terms of (5):

$A_{G,1}=b_{1}\left( \frac{1}{b_{2}}\left( A_{G,2}-e_{2} \right) \right)+e_{1}=\frac{b_{1}}{b_{2}}A_{G,2}-\frac{b_{1}}{b_{2}}e_{2}+e_{1}$(6)

When estimated in the first stage of an instrumental variable regression, the predicted values Â_G,1_ = γA_G,2_b (γ = b_1_/b_2_) used in the second stage only consider the correlation between the scores.

$y=\alpha+\betaÂ_{G,1}+\varepsilon$(7)

The roles of the two PGS can also be reversed in which the estimated equation becomes.

$y=\alpha+\betaÂ_{G,2}+\varepsilon$(8)

To our knowledge, it has not been studied if one variable is better suited to use as the instrumental variable than the other. If it is assumed that the correlation of one PGS with the unobserved polygenic load is stronger than the other, b_1_ > b_2_ , implying that e_1_ < e_2_, then it appears that A_PGS,1_ should be instrumented with A_PGS,2_. We performed simulations to test this.

**Simulations**

Denote the polygenic load for ADHD with G, two polygenic scores of G with G_1_ and G_2_. These are assumed to be normally distributed and random draws are generated using a multivariate normal distribution using mean vector µ = (0, 0, 0), and (symmetric) variance-covariance matrix:

|  | G | G_1_ | G_2_ |
| --- | --- | --- | --- |
| G | 1 | ρ_1_ | ρ_2_ |
| G_1_ |  | 1 | ρ_1,2_ |
| G_2_ |  |  | 1 |

ρ denotes the true covariances. These are drawn from a uniform distribution where ρ_G,1_ ∼ U (0.05, 0.095), ρ_G,2_ ∼ U (0.01, 1 − ρ^2^_G,1_), thereby G_1_ is always more strongly correlated with G. In addition, an error distribution of e ∼ N (0, 1) in G_1_ and G_2_ was added in the measurements of G.

$G_{1}=\rho_{G,1}G+e_{1}$(9)

$G_{2}=\rho_{G,2}G+e_{2}$(10)

ADHD is then defined as a function of genetic (G) and environmental (E) factors we get:

ADHD = G + E (11)

Given G,E ∼ N(0,1), about 50 percent of the variation in ADHD is explained by genetic and environmental factors each. Subsequently, the outcome is defined as:

$$y=a+b_{1}G+b_{2}E+e$$

where ɑ = 1, b_1_ = b_2_ = -0.3, and e ∼ N(0,1), a normally distributed error term.

A total of 100 correlations ρ_G,1_ were drawn, and for each of these 100 correlations were drawn for ρ_G,2_, thereby generating 10000 different covariance matrices. The R-package MASS (4) was then used to generate a dataset with 1000 observations from the distributions described by the covariance matrix outlined above.

In each dataset linear regression and two-stage least squares were used to estimate associations between G_1_, G_2_ and y. Figure 1 shows the result. Results from IV analyses have been restricted to observations where the first stage regression is significant (p-value < 0.05). The upper panel shows the crude models and illustrates the downward bias in the ordinary linear regression as the associations are closely aligned towards zero and that this happens regardless of how strong the correlation with the polygenic load is. The IV analyses are more likely to align with the true association (black line), and perform better the higher the correlation with the polygenic load is. As indicated by the green area, when the polygenic score that correlates higher with the polygenic load (G_1_) is instrumented by the one with the weaker correlation (G_2_), the distribution is more closely aligned towards the true association. The lower panel shows the effect of including ADHD. Since ADHD encompass G, all associations centers on zero (which they should). However, the IV analysis is more likely to yield devations from zero compared to ordinary analyses.

| Figure 1. Simulation results: Density plots | |
| --- | --- |
|  | Restricted: ρ_1,2_ = (0.2, 0.8) and ρ_2_ > 0.2 |
| Crude model: $y=\beta_{0}+\beta_{1}G_{1,2}+\varepsilon$ | |
| 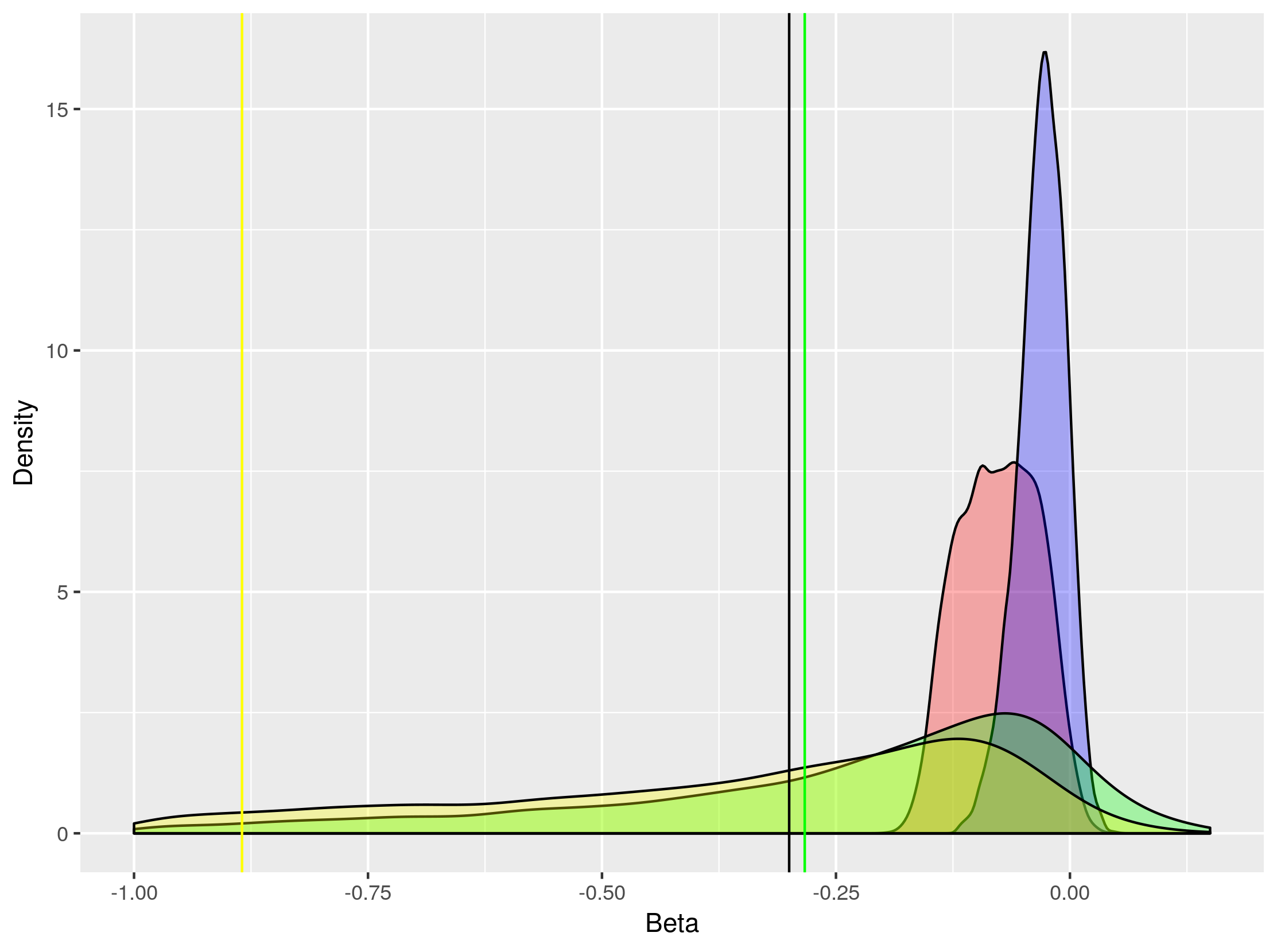 | 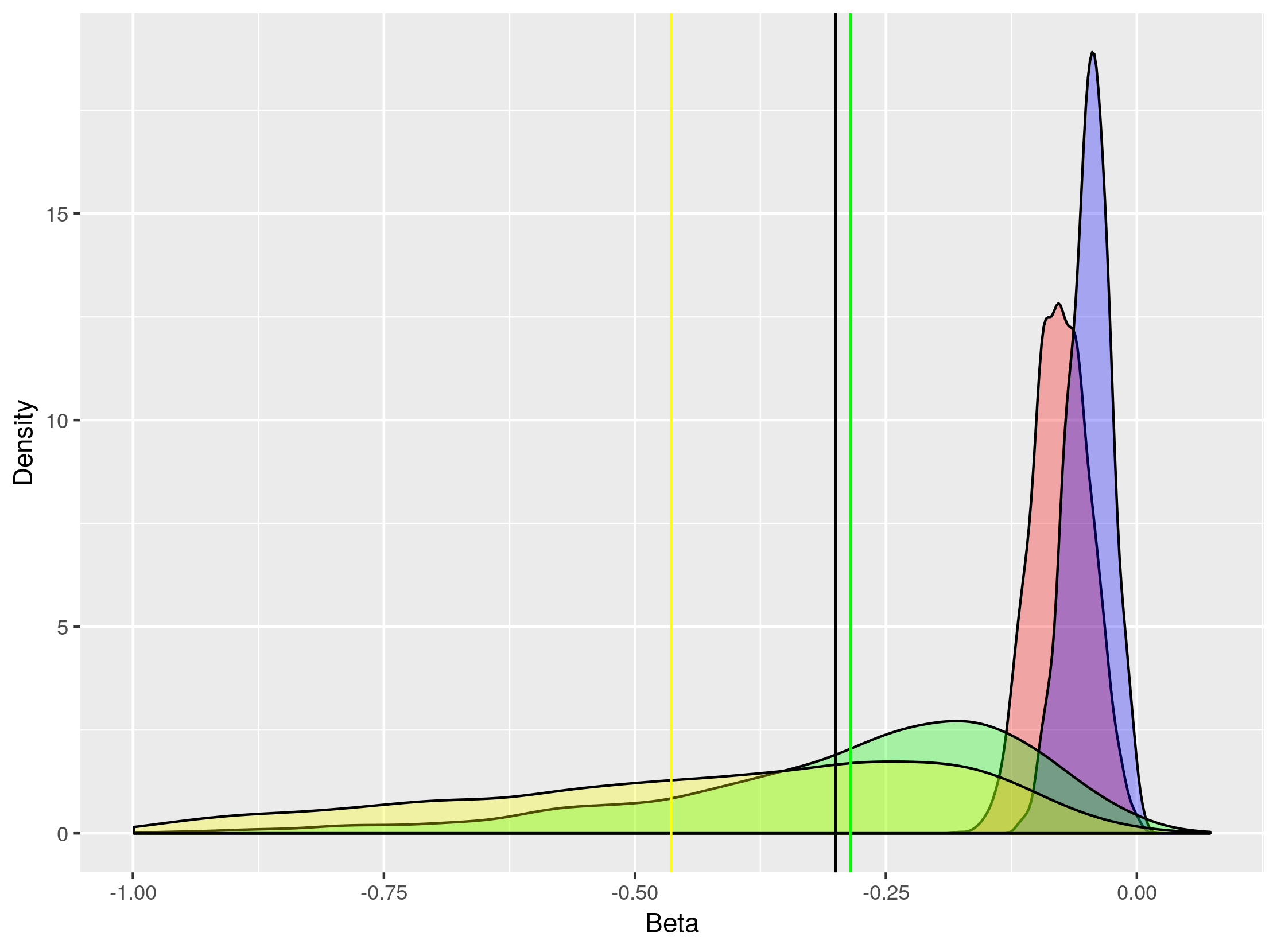 |
| Adjusted for ADHD: $y=\beta_{0}+\beta_{1}ADHD+\beta_{2}G_{1,2}+\varepsilon$ | |
| 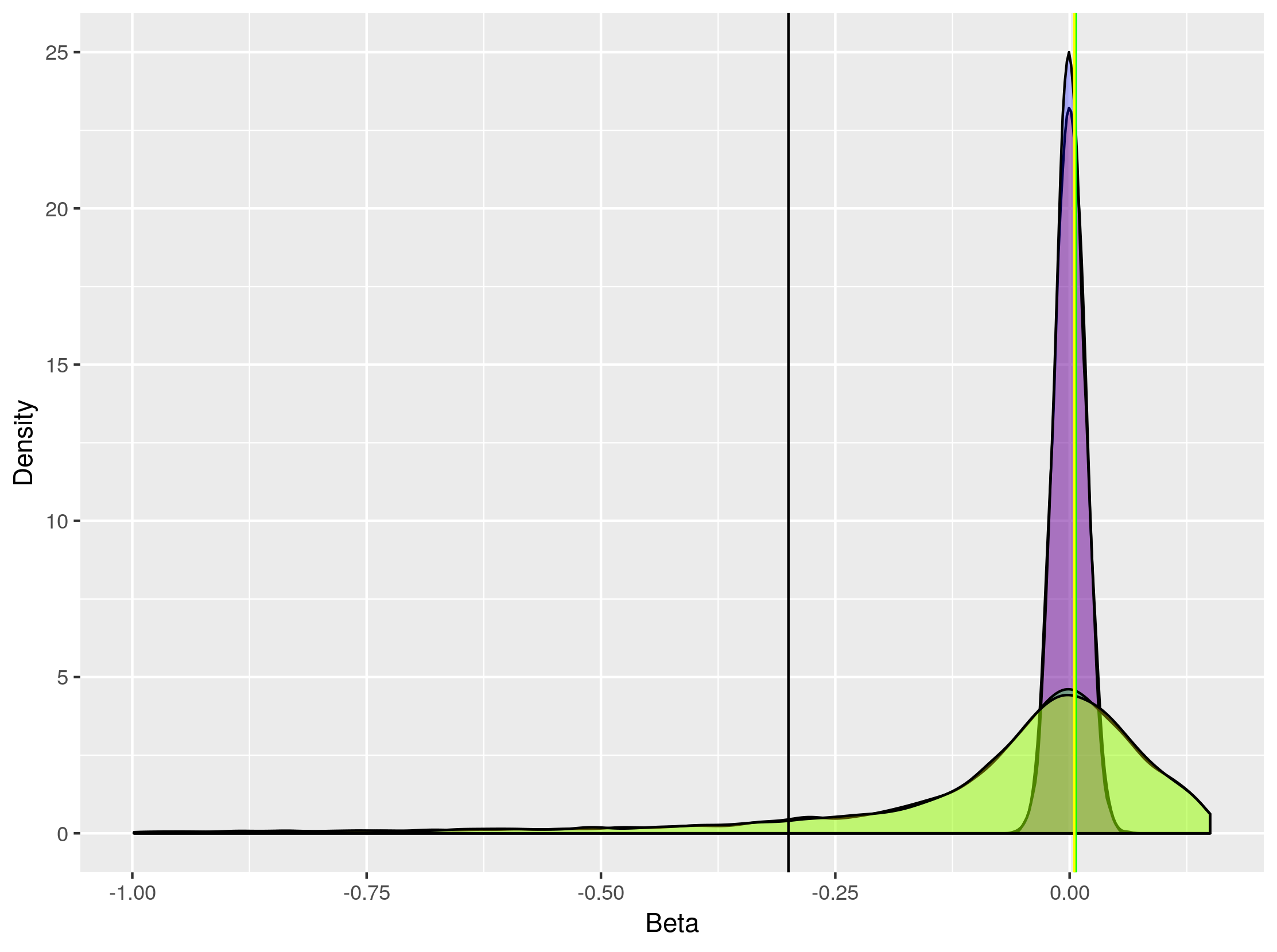 | 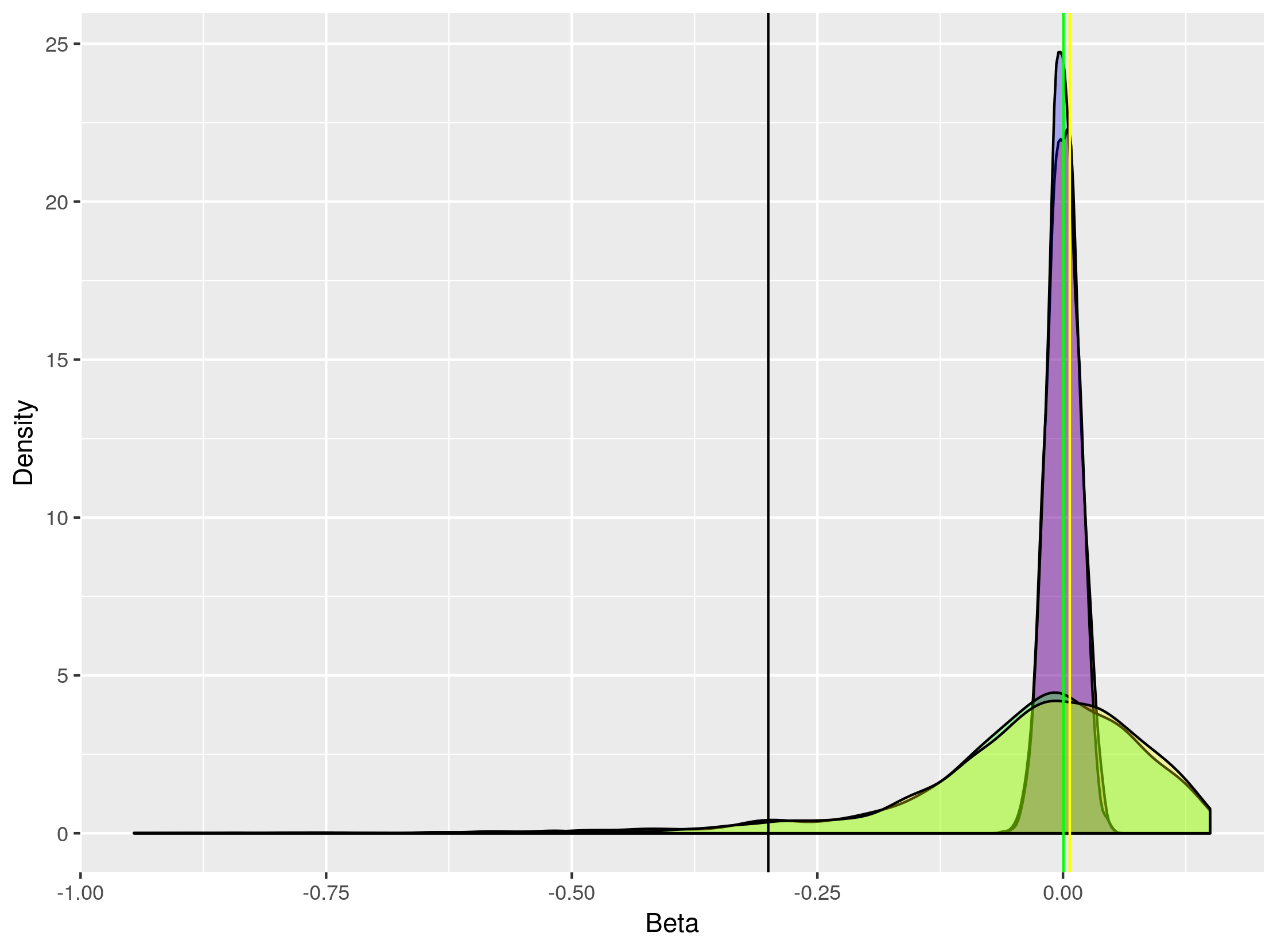 |
| Red: β from OLS for G_1_ . Blue: β from OLS for G_2_ . Green: β from IV-analysis, G_1_ instrumented by G_2_ . Yellow: β from IV-analysis, G_2_ instrumented by G_1_ . Green and yellow vertical lines indicate means of the estimated effect of G using the IV method. Black vertical lines indicate the true effect of G on y. Restricted: In the right panels results are restricted to PGS with covariance in the range 0.2 to 0.8 with G and the the second PGS with minimum correlation with G of 0.2. | |

**Table S1: Results from first stage regressions in the instrumental variable analyses**

The validity of the instrumental variable analyses depend on that the two PGS are strongly correlated with each other. The table below shows the results from the first stage regression where the instrumented variable (“Outcome” in the table below) is regressed on the designated instrumental variable (“Exposure” in the table below). The R-package clubSandwich was used to calculate the F-statistics.

|  |  | Model | | | | |
| --- | --- | --- | --- | --- | --- | --- |
| PGS | | β standard deviations; p-value (F-statistic) | | | | |
| Outcome | Exposure | Crude | ADHD symptoms | EA-PGS | ADHD symptoms + EA-PGS | Within family |
| ADHD-PGS | ADHS-PGS | 0.063; 8e-05(15.56) | 0.060; 0.00015(14.39) | 0.048; 0.0021(9.49) | 0.047; 0.0027(8.99) | 0.027; 0.427(NA) |
| ADHS-PGS | ADHD-PGS | 0.063; 7.1e-05(15.77) | 0.061; 0.00013(14.58) | 0.050; 0.002(9.58) | 0.049; 0.0026(9.07) | 0.028; 0.428(NA) |
| All associations are adjusted for sex, the linear effect of graduation year and the first five principal components. PGS: Polygenic score. ADHD symptoms: Model adjusted for the mean symptoms on A-TAC’s attention and impulsiveness scale. EA-PGS: Model adjusted for the PGS for educational attainment. Within family: Model adjusted for shared familial factors through a dizygotic twin comparison. Standard errors have been clustered on family. | | | | | | |

**Table S2: Association between polygenic scores for ADHD and phenotypic symptoms, and clinical diagnosis of ADHD**

**Definition of clinical ADHD**

Clinically diagnosed ADHD was identified using the National Patient Register (NPR) that includes inpatient care since 1970, and outpatient care since 2001, and the Prescribed Drug Register (PDR) which contains dispensed medications using Anatomical Theurapeutic Chemical (ATC) codes. Clinical ADHD was defined as diagnosis of ADHD in the NPR, code F90 in International Classification of Diseases version 10, and/or a dispensed medication for ADHD (ATC code) amphetamines (N06BA01, N06BA02), methylphenidate (N06BA04), and atomoxetine (N06BA09) in the PDR. ADHD was considered in terms of lifetime presence (ever vs. never diagnosed/medicated) in accordance with prior research using Swedish registers (5). This definition shows a high correlation with ADHD symptoms as measured by the A-TAC questionnaire used in

the current sample (6).

|  |  | **Polygenic scores** | |
| --- | --- | --- | --- |
|  |  | ADHD-PGS | ADHS-PGS |
|  |  | **Ordinary linear/logistic regression** | |
| **ADHD phenotype** | **Model** | **β standard deviations; p-value** | |
| Attention/impulsiveness | Crude | 0.077; 1.6e-07 | 0.040; 0.0045 |
|  | EA-PGS | 0.062; 2.5e-05 | 0.033; 0.018 |
| Attention | Crude | 0.062; 2.2e-05 | 0.038; 0.0058 |
|  | EA-PGS | 0.062; 2.5e-05 | 0.033; 0.018 |
| Impulsiveness | Crude | 0.076; 1.2e-07 | 0.033; 0.020 |
|  | EA-PGS | 0.062; 2.5e-05 | 0.033; 0.018 |
|  |  | Odds-ratio; p-value | |
| Clinical diagnosis | Crude | 1.521; 1.4e-05 | 1.066; 0.485 |
|  | EA-PGS | 1.420; 0.00031 | 1.035; 0.706 |
|  |  | Instrumental variable analysis | |
|  |  | Instrumental variable | |
|  |  | ADHS-PGS | ADHD-PGS |
|  |  | β standard deviations; p-value | |
| Attention/Impulsiveness | Crude | 0.630; 0.015 | 1.220; 0.0013 |
|  | EA-PGS | 0.672; 0.050 | 1.220; 0.012 |
| Attention | Crude | 0.599; 0.018 | 0.986; 0.0032 |
|  | EA-PGS | 0.631; 0.059 | 0.916; 0.025 |
| Impulsiveness | Crude | 0.518; 0.036 | 1.193; 0.0014 |
|  | EA-PGS | 0.560; 0.085 | 1.268; 0.010 |
|  |  | Odds-ratio; p-value | |
| Clinical diagnosis | Crude | 1.549; 1.7e-05 | 1.111; 0.270 |
|  | EA-PGS | 1.397; 0.012 | 1.044; 0.700 |
| In the instrumental variable analyses, the instrumental variable is the PGS predicting the exposure PGS in the first stage. For example, in the column "ADHS-PGS" (PGS for ADHD symptoms) the ADHD-PGS is regressed on the ADHS-PGS in the first stage, and in the second stage the predicted values of the ADHD-PGS are used in estimating the association with each phenotype. For estimates in the column "ADHS-PGS" their roles are reversed. All associations are adjusted for female sex, the linear effect of graduation year, and the first five principal components. Standard errors have been clustered on families. | | | |

**Figure S1: Supplementary variable analysis of the association between PGS for ADHD and school performance**

In this instrumental variable analysis the role of the polygenic scores have been reversed, and the polygenic score for ADHD diagnosis is used as an instrument for the polygenic score for ADHD symptoms.

| 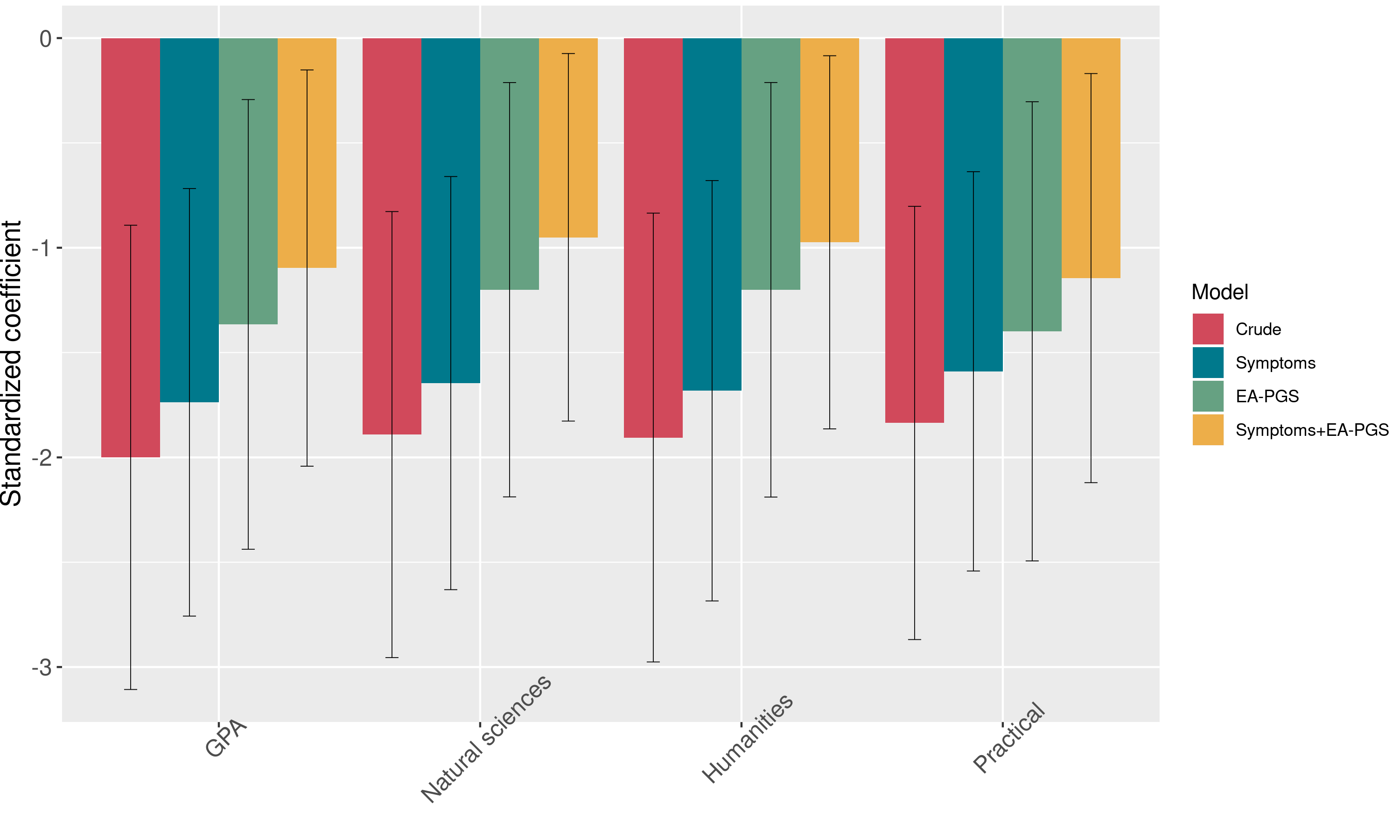 |
| --- |
| All associations adjusted for sex, the linear effect of graduation year, and the first five principal components. GPA: Grade point average. Natural sciences: GPA in biology, chemistry, mathematics, and physics. Humanities: GPA in civics, English, geography, history, religion, and Swedish. Practical: GPA in arts, crafts, home and consumer studies, music, sports, and technology. |

**Figure S2: Association between PGS for ADHD and GPA stratified on sex**

| **Females** |
| --- |
| 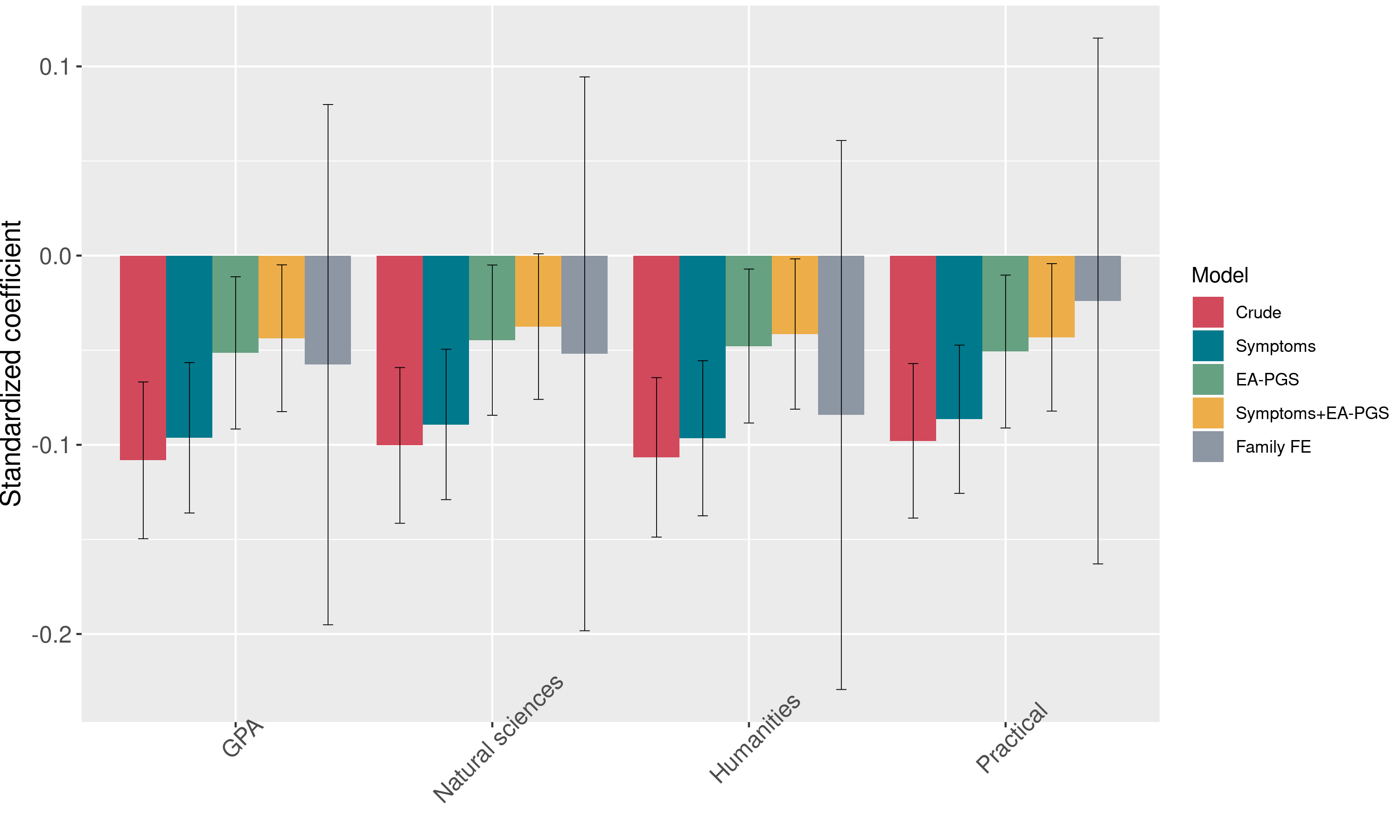 |
| **Males** |
| 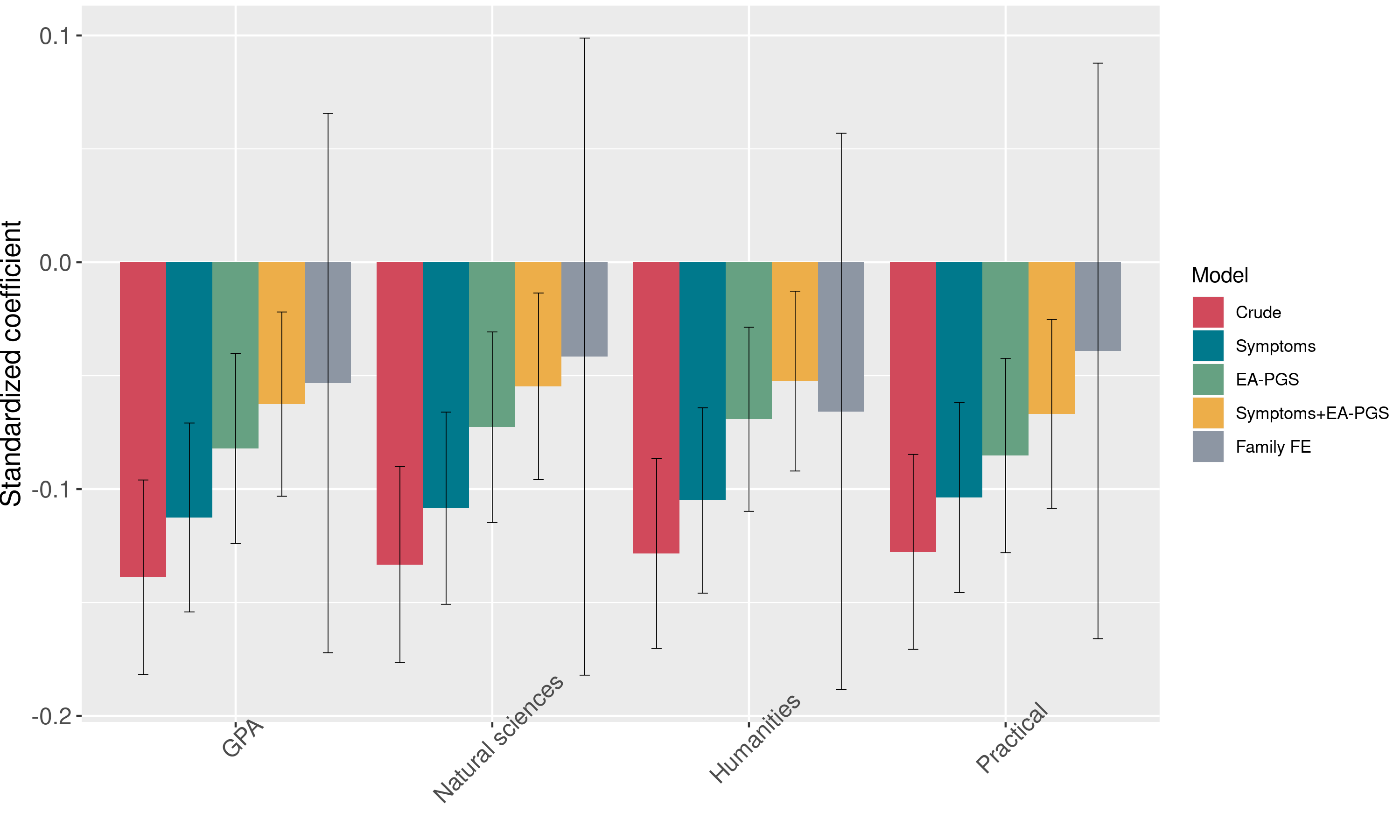 |
| All associations adjusted for the linear effect of graduation year, and the first five principal components. GPA: Grade point average. Natural sciences: GPA in biology, chemistry, mathematics, and physics. Humanities: GPA in civics, English, geography, history, religion, and Swedish. Practical: GPA in arts, crafts, home and consumer studies, music, sports, and technology. |

**Figure S3: Association between PGS for ADHD and symptom subtype**

| 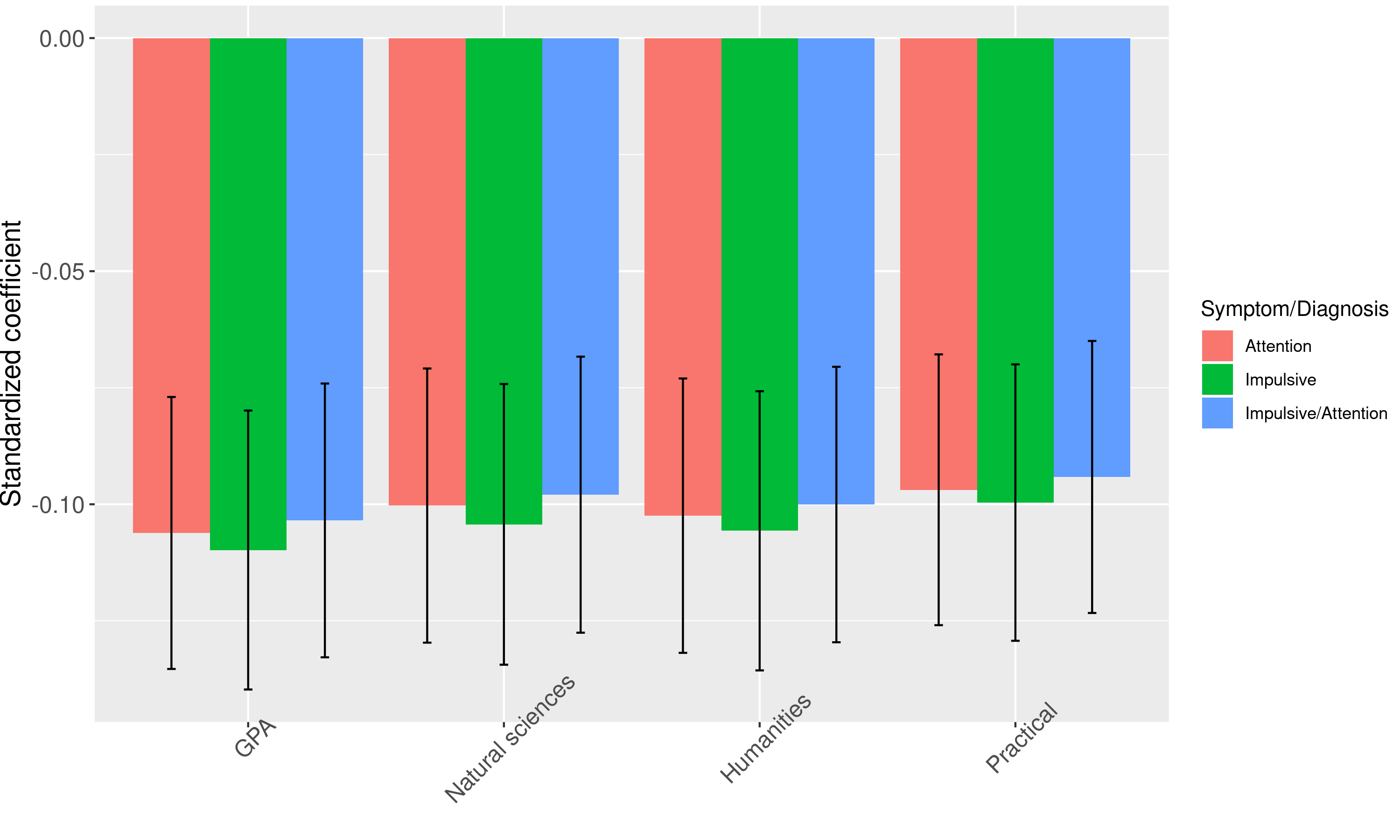 |
| --- |
| All associations adjusted for the linear effect of graduation year, and the first five principal components. GPA: Grade point average. Natural sciences: GPA in biology, chemistry, mathematics, and physics. Humanities: GPA in civics, English, geography, history, religion, and Swedish. Practical: GPA in arts, crafts,home and consumer studies, music, sports, and technology. |

**Figure S4: Associations between PGS for ADHD and individual subjects**

| **Ordinary linear regression** |
| --- |
| 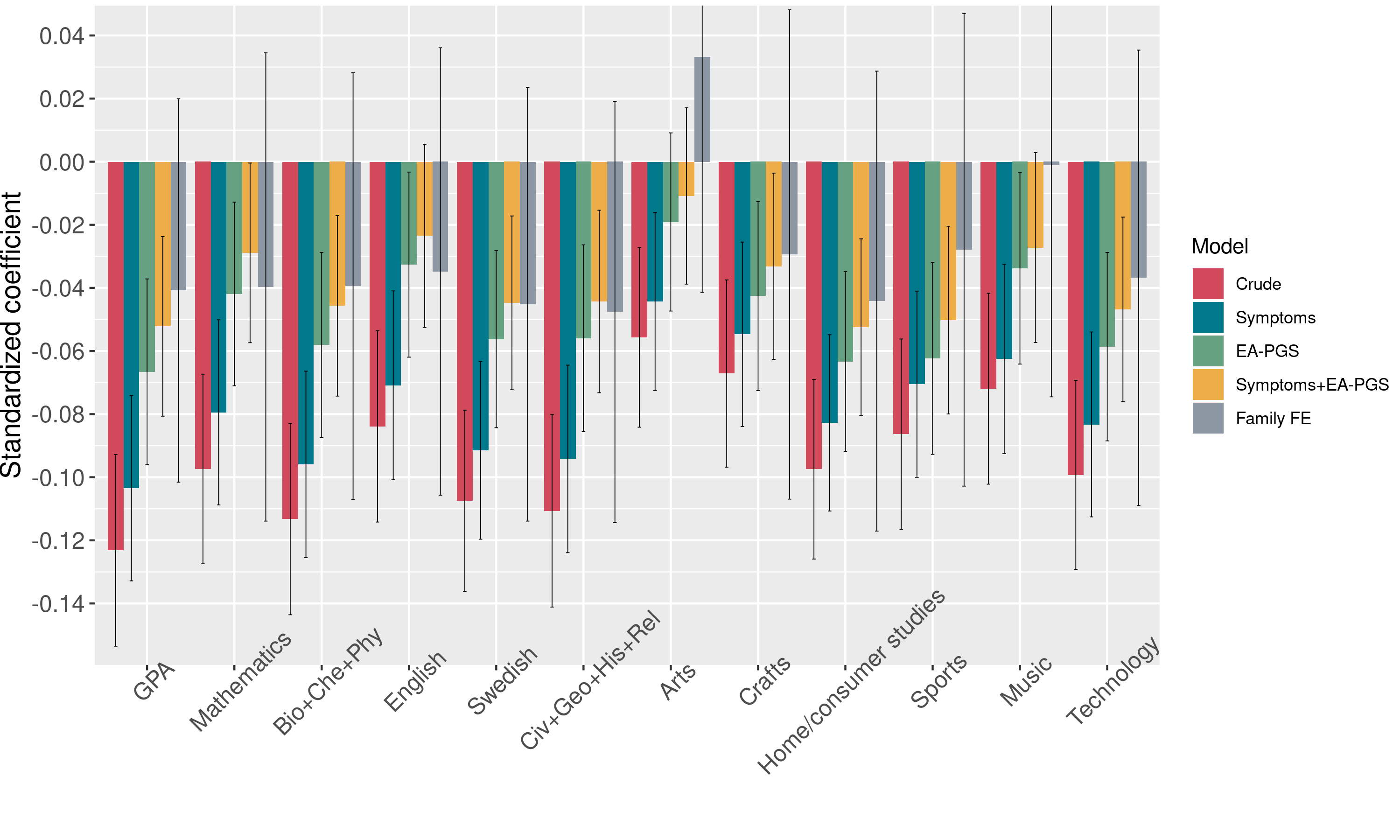 |
| **Instrumental variable regression** |
| 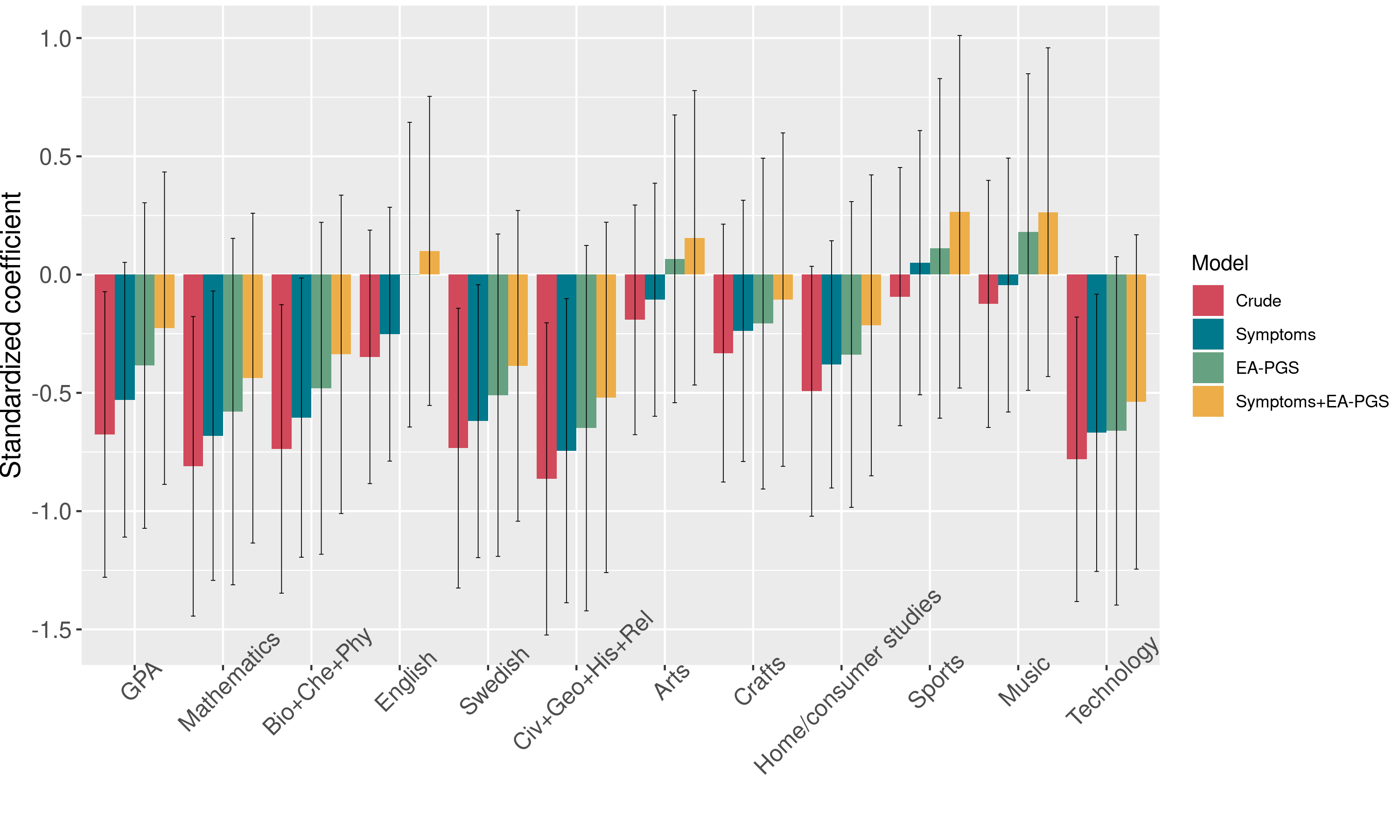 |
| All estimates adjusted for sex, the linear effect of graduation year, and the first five principal components. In the instrumental variable regression, the polygenic score for ADHD symptoms is used as an instrument for the polygenic score for ADHD diagnosis. Standard errors were clustered on families. GPA: Grade point average. Bio+Che+Phy: Average grade in biology, chemistry, and physics. Civ+Geo+His+Rel: Civics, geography, history, and religion. Family FE: Family fixed-effects, i.e., a within twin pair association. Sex was the only covariate in this model. As the criteria for an IV analysis was not fulfilled for the family fixed-effect been omitted from this plot (lower panel). |

**Figure S5: Association between PGS for ADHD and differences between subject category GPA**

| **Ordinary linear regression** |
| --- |
| 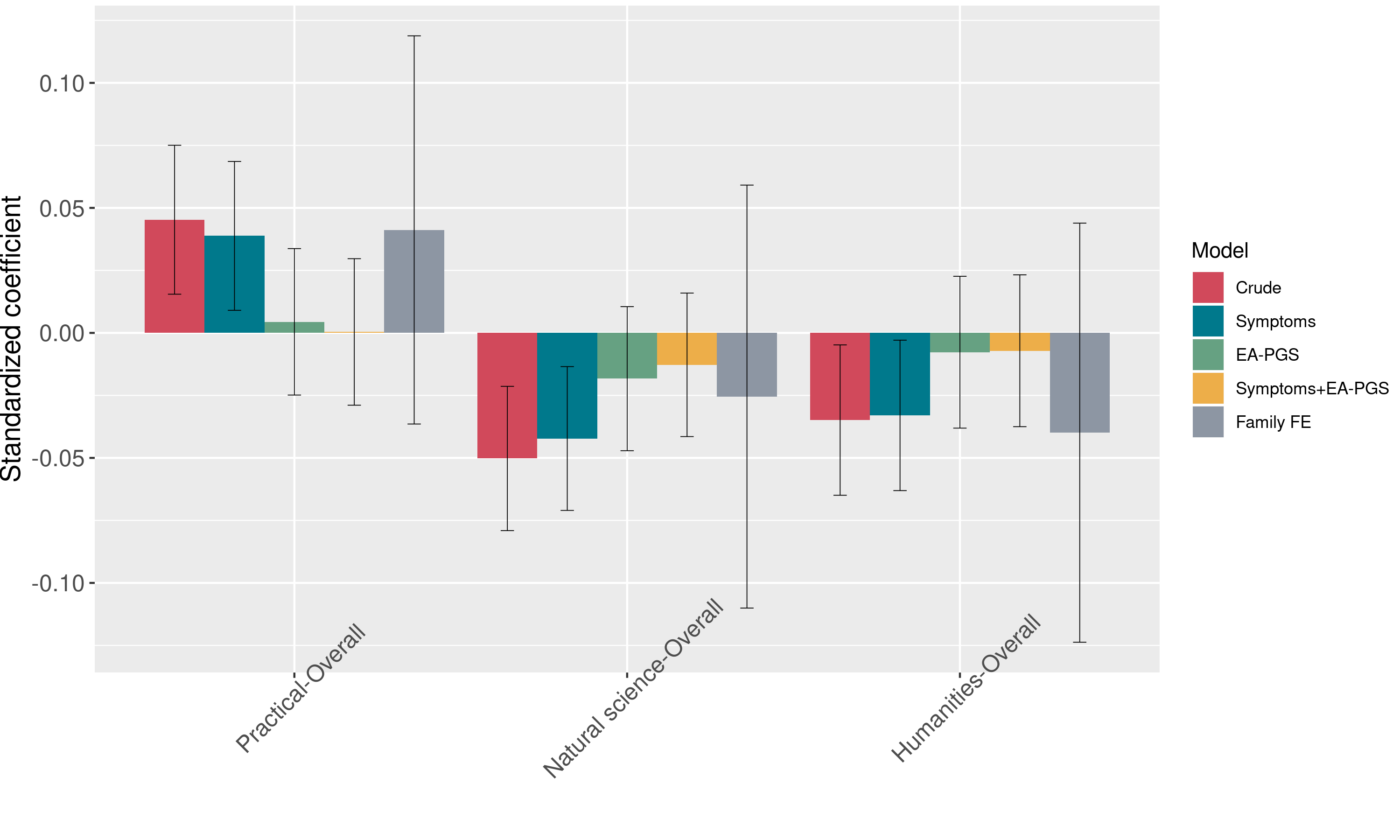 |
| **Instrumental variable regression** |
| 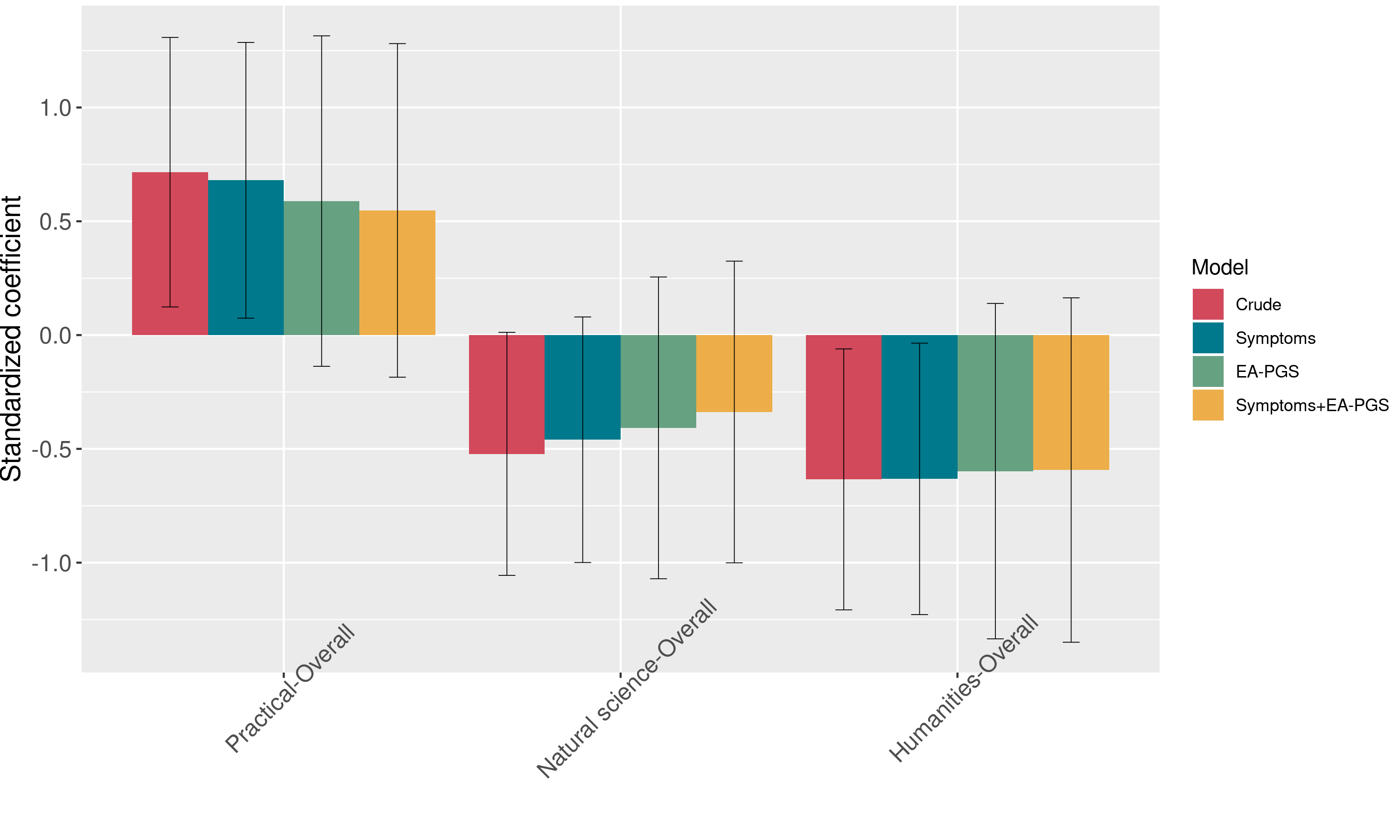 |
| All estimates adjusted for sex, the linear effect of graduation year, and the first five principal components. In the instrumental variable regression, the polygenic score for ADHD symptoms is used as an instrument for the polygenic score for ADHD diagnosis. Standard errors were clustered on families. Natural-Humanities: Grade point average (GPA) difference between natural science and humanities subject blocks. Practical-Natural: GPA difference between practical and natural science subjects. Practical-Humanities: GPA difference between practical and humanities subject blocks. Family FE: Family fixed-effects, i.e., a within twin pair association. Sex was the only covariate in this model. As the criteria for an IV analysis was not fulfilled for the family fixed-effect model (within twin-pair), these estimates has been omitted from this plot (lower panel). |

**References**

1. Ramstedt, K. National assessment and grading in the Swedish school system. https://www.skolverket.se/download/18.6bfaca41169863e6a655954/1553958924171/pdf1524.pdf (2005).

2. Wooldridge, J. M. Econometric analysis of cross section and panel data. (MIT Press, 2010).

3. DiPrete, T. A., Burik, C. A. P. & Koellinger, P. D. Genetic instrumental variable regression: Explaining socioeconomic and health outcomes in nonexperimental data. Proceedings of the National Academy of Sciences 115, E4970–E4979 (2018).

4. Venables, W. N., Ripley, B. D. & Venables, W. N. Modern applied statistics with S. (Springer, 2002).

5. Sun, S. et al. Association of Psychiatric Comorbidity With the Risk of Premature Death Among Children and Adults With Attention-Deficit/Hyperactivity Disorder. JAMA Psychiatry (2019) doi:10.1001/jamapsychiatry.2019.1944.

6. Larsson, H. et al. Risk of bipolar disorder and schizophrenia in relatives of people with attention-deficit hyperactivity disorder. Br J Psychiatry 203, 103–106 (2013).

1. Swedish National Agency for Education, “Swedish grades”, accessed 2021-06-28: https://www.skolverket.se/download/18.47fb451e167211613ef398/1542791697007/swedishgrades_bilaga.pdf [↑](#footnote-ref-1)
